# An approved *in vitro* approach to preclinical safety and efficacy evaluation of engineered T cell receptor anti-CD3 bispecific (ImmTAC) molecules

**DOI:** 10.1371/journal.pone.0205491

**Published:** 2018-10-15

**Authors:** Jane Harper, Katherine J. Adams, Giovanna Bossi, Debbie E. Wright, Andrea R. Stacey, Nicole Bedke, Ruth Martinez-Hague, Dan Blat, Laure Humbert, Hazel Buchanan, Gabrielle S. Le Provost, Zoe Donnellan, Ricardo J. Carreira, Samantha J. Paston, Luise U. Weigand, Martina Canestraro, Joseph P. Sanderson, Sophie Botta Gordon-Smith, Kate L. Lowe, Karolina A. Rygiel, Alex S. Powlesland, Annelise Vuidepot, Namir J. Hassan, Brian J. Cameron, Bent K. Jakobsen, Joseph Dukes

**Affiliations:** 1 Immunocore Ltd, Abingdon, Oxford, United Kingdom; 2 Adaptimmune, Oxford, United Kingdom; Jackson Laboratory, UNITED STATES

## Abstract

Robust preclinical testing is essential to predict clinical safety and efficacy and provide data to determine safe dose for first-in-man studies. There are a growing number of examples where the preclinical development of drugs failed to adequately predict clinical adverse events in part due to their assessment with inappropriate preclinical models. Preclinical investigations of T cell receptor (TCR)-based immunotherapies prove particularly challenging as these biologics are human-specific and thus the conventional testing in animal models is inadequate. As these molecules harness the full force of the immune system, and demonstrate tremendous potency, we set out to design a preclinical package that would ensure adequate evaluation of these therapeutics. Immune Mobilising Monoclonal TCR Against Cancer (ImmTAC) molecules are bi-specific biologics formed of an affinity-enhanced TCR fused to an anti-CD3 effector function. ImmTAC molecules are designed to activate human T lymphocytes and target peptides within the context of a human leukocyte antigen (HLA), thus require an intact human immune system and peptidome for suitable preclinical screening. Here we draw upon the preclinical testing of four ImmTAC molecules, including IMCgp100, the first ImmTAC molecule to reach the clinic, to present our comprehensive, informative and robust approach to *in vitro* preclinical efficacy and safety screening. This package comprises a broad range of cellular and molecular assays using human tissues and cultured cells to test efficacy, safety and specificity, and hence predict human responses in clinical trials. We propose that this entirely *in vitro* package offers a potential model to be applied to screening other TCR-based biologics.

## Introduction

The immune system, when harnessed, is the most powerful weapon we have against cancer. Aberrant tumour cells, however, are capable of immune evasion. Extensive efforts over the last few decades have led to the emergence of diverse immunotherapeutic strategies aimed at re-engaging immune cells to enhance the recognition and elimination of tumour cells [1, 2]. Therapies that activate the immune system, such as IL-2, TNFα or monoclonal antibodies against immune checkpoint molecules CTLA-4 and PD-1, have demonstrated long-lasting clinical benefit [3]. Immune checkpoint modulators have shown particular promise, functioning to release the brakes on the immune system and restore T cell cytotoxic anti-tumour activity [4]. Additionally, antigen-targeted approaches in the form of monoclonal antibodies, bispecific molecules, chimeric antigen receptor (CAR) T cells or T cell receptor (TCR)-based therapies have shown varied success against specific cancers [2, 5].

Amongst the TCR-based therapeutics are the Immune Mobilising Monoclonal TCRs Against Cancer (ImmTAC) molecules, which comprise a soluble affinity enhanced TCR fused to an anti-CD3 single chain variable fragment (scFv). ImmTAC molecules engage peptides presented in the context of human leukocyte antigen (HLA), thus offering exquisite specificity. ImmTAC molecules have been shown to re-direct endogenous T cells specifically to tumour cells presenting target peptide:HLA [6].

Therapies that use T cells, be they adoptively transferred or recruited through the introduction of bispecific biologics such as ImmTAC molecules, have demonstrated tremendous potency, which, if misdirected, have the potential to cause serious toxicities [7]. Several fatal incidences following adoptive cell therapy have been reported, highlighting the need for continual re-assessment of preclinical evaluation strategies [7–9]. A key challenge in the preclinical evaluation of T cell-based therapeutics, is the species-specific nature of the cellular and protein components of the human immune system. In the case of ImmTAC molecules, both ends of the bispecific protein are exquisitely human-specific, with the TCR engaging human peptide:HLA (pHLA) and the anti-CD3 domain activating only human T lymphocytes.

Strategies used to preclinically assess the risk of T cell-based therapies include species-specific surrogate molecules (as opposed to the human-specific clinical molecule) tested in animal models or human-specific molecules tested in humanised mouse models or non-human primates. Some biologics, including monoclonal antibodies or bispecific reagents, have been successfully tested in animal models using surrogate counterparts, while many others have suffered from poor predictability [10–16].

Animal models are not deemed suitable for ImmTAC testing for a number of reasons: (i) the human immune system differs markedly from other species and hence immune responses observed in animals may not predict human responses, (ii) proteomes across species show limited overlap and may fail to present the same antigens, (iii) the TCR portion of a surrogate ImmTAC molecule would differ in specificity and binding profile to the human TCR.

The absence of HLA is a particular hurdle for the testing of TCR-based therapies in mouse models, given the exquisite specificity of these molecules to the human pHLA complex [17]. Mice engineered to express HLA have been developed but still present murine peptides and lack the human peptide processing machinery [18]. It is estimated that ~40% of the mouse MHC Class I peptidome is identical to humans (our unpublished data), therefore the remaining 60% of human pHLA molecules would not be presented for cross-reactivity testing and thus such a model would be uninformative. Preclinical assessment of immunotherapeutic molecules in non-human primates has also posed a significant challenge due to poor translation to human safety [19, 20]. For example, comparison of immunogenicity (measured by the formation of anti-drug antibodies (ADAs)) across a panel of clinically-relevant monoclonal antibodies (mAbs) showed only 59% correlation between non-human primates and humans [21].

Efforts to improve existing animal models have yet to successfully recapitulate the full complexity of the human immune system and proteome [20, 22]. The Wistar meeting on future prospects of preclinical studies of melanoma highlighted that a fully functional human immune system is indispensable to effectively study immune modulatory therapies [23]. Guidance set in relation to preclinical testing of new drug candidates at the International Conference on Harmonization ICHS6 and ICHS9 outlined that preclinical studies should be carried out in relevant species in which the test material is pharmacologically active. Where no such species are available, *in vitro* assessments should be considered. Furthermore, the selection of a starting dose based on MABEL (Minimal Anticipated Biological Effective Level) was advised to be considered as an alternative to NOAEL (No Observable Adverse Effect Levels).

A growing body of evidence suggests that human-specific *in vitro* studies can generate informative, predictive data [24]. A range of *in silico* and *in vitro* assays are routinely performed to preclinically assess toxicity, specificity and cross-reactivity of drug candidates [25]. Pharmacological activity of a test molecules can be assessed using clinically relevant *in vitro* and *ex vivo* models that assess cell activation, suppression, cytokine production and immune modulation, and incorporate human cells and tissues [25].

Here we describe how the understanding of an ImmTAC molecules mechanism of action has enabled the development of a systematic package of *in vitro* assays to assess the potency and specificity (safety) of this novel biological entity. This package involves (i) human cell testing utilising a wide-range of normal and cancer cells and (ii) molecular analysis of the ImmTAC peptide binding motif to identify cross-reactive ‘mimic’ peptides in the genome. Collectively these data inform on the therapeutic potential, specificity and safety considerations, as well as a clinical starting dose for an ImmTAC molecule [26]. Augmentation of the preclinical package to further improve safety predictions and mitigate the risk of off-target effects have been incorporated as a result of our increased understanding of TCR cross-reactivity.

To exemplify the application of this paradigm, this report draws on the preclinical testing of four example ImmTAC molecules, including IMCgp100, the first ImmTAC molecule to reach the clinic. We propose that this entirely *in vitro* preclinical assessment package represents a potential paradigm shift in the approach to preclinical assessment of TCR-based therapies by providing a more physiologically relevant substitute for traditional *in vivo* preclinical testing.

## Materials and methods

### Cell lines and cell culture

Cell lines used in this study were grown according to the manufacturers’ instructions (S1 Table). CAMA-1 cells express a low level of HLA-A2 that result in very low surface levels, thus this cell line was transduced in-house with a lentivirus containing HLA-A2 and beta 2-microglobulin (CAMA-1 A2b2m) for assays with ImmTAC-nybr1. Cell line authentication and mycoplasma testing were routinely carried out by the LGC Standards Cell line Authentication Service (www.lgcstandards.com) and Mycoplasma Experience Ltd (www.mycoplasma-exp.com), respectively. Unless stated, peripheral blood mononuclear cells (PBMCs) were obtained from healthy volunteers. Where indicated, PBMCs were obtained from a patient with melanoma, kindly provided by Neil Steven from University of Birmingham. The Oxford A REC approved protocols 13/SC/0226 (Immunocore study protocol number IMCres02) was used to obtain written consent for all blood donations and was fully approved by the National Research Ethics Committee (NRES) South Central.

### ImmTAC molecules

ImmTAC molecules used in this study were (i) ImmTAC-gp100, recognising a gp100 peptide in the context of HLA-A*02, referred to in the clinic as IMCgp100 [6]; (ii) ImmTAC-mageA3, recognising a MAGE-A3 peptide in the context of HLA-A*01[27, 28]; (iii) ImmTAC-nybr1, recognising an NYBR1 peptide in the context of HLA-A*02 and (iv) ImmTAC-nyeso, recognising an NY-ESO-1 peptide in the context of HLA-A*02, referred to in previous publications as NYCAN0 [29]. ImmTAC molecules were generated as previously described [6].

### Quantitative real-time PCR (qPCR)

Total RNA was extracted from cells using RNeasy mini kit (Qiagen). RNA quantity, purity and integrity were assessed with Nanodrop 2000 and Tape Station 4200 (Applied Biosystems). RNA was reverse-transcribed into cDNA using the iScript Advanced cDNA Synthesis kit (Bio-Rad). Hydrolysis probe-based qPCR assays custom designed for gp100 (forward 5’-TTCTGCACCAGATACTGAAGGGT-3’; reverse 5’-GATAAGCTGGGTGCTGACCACT-3’; probe 5’-[FAM]-ACATACTGCCTCAATGTG-[NFQ-MGB]-3’) (TaqMan, Life Technologies) were used with the QuantiTect Probe PCR kit (Qiagen). qPCR reactions were performed in duplicate (Quantstudio 6 real-time PCR system, Life Technologies). A standard curve of serial 1:10 dilutions of a known copy number of DNA template were analysed in parallel and the number of transcripts quantified using absolute quantification. Data were normalised to two reference genes: RPL32 and HPRT1 and the data presented as Normalised Relative Quantity (NRQ). NRQ = (RQ target gene/geometric mean RQ housekeeping genes) x 10^4^, RQ = Efficiency-CT.

### Flow cytometry

Cells were incubated with anti-HLA-A2-FITC antibody (BioLegend, cat. 343304) or anti-IgG2b-FITC isotype control antibody (BioLegend, cat. 401206) diluted at 1:50 in PBS for 30 min at 4°C. Cells were washed and resuspended in 50 μl of FACS buffer (PBS + 0.5% BSA + 4 Mm EDTA). Data was acquired using BD Accuri C6 platform (BD Biosciences) and represented as relative abundance in mean fluorescence intensity (MFI) relative to isotype/unstained ratio.

### IncuCyte assay

Killing assays were carried out using the IncuCyte FLR-Platform (EssenBioSciences) as described previously [30]. Briefly, target cells and effector cell CD8+ T effector cells were plated in flat bottomed 96-well plates with decreasing concentrations of ImmTAC-gp100 (0 pM -1000 pM). Cellular apoptosis was detected using NucView reagent (Biotium). Images were taken every 2 hours for 52 hours using CellPlayer. Vybrant DyeCycle Green Stain (ThermoFisher Scientific) was added at the end of each experiment (when object counts/mm2 reached a plateau) to determine the apoptotic index, measured as a ratio of apoptotic cells to the total number of cells in a field of view.

### IFNγ ELISpot

IFNγ ELISpot assays were performed according to the manufacturer’s instructions (BD Biosciences). Briefly, target cells were plated at ~5x10^4^ cells/per well and incubated with PBMC effector cells at a donor-dependant density. For alloreactivity assays, target cells were sourced to cover an extensive panel of HLA-types including all HLA-types that exist at frequencies of >10% of the target population. Cells were integrated from multiple sources, all of know HLA-type, including a range of; B cells isolated from frozen PBMCs from healthy donors, lymphoblastoid cell lines (Leiden University) and cancer cell lines (ATCC). ImmTAC molecules were added at the indicated concentration and plates were incubated overnight at 37°C/5% CO_2_. IFNγ-release was quantified using the BD ELISpot reader (Immunospot Series 5 Analyzer, Cellular Technology Ltd).

### LDH release killing assay

CytoTox 96 Non-Radioactive Cytotoxicity Assays were performed according to the manufacturer’s instructions (Promega). Briefly, Ag+ and Ag- target cells were seeded at ~1x10^4^ cells/per well and incubated with PBMCs obtained from healthy donors. ImmTAC molecules were added at the indicated concentrations and plates were incubated at 37°C/5% CO_2_ for 24h. Results were collected using the VersaMax microplate reader.

### Cytokine analysis

Whole blood of healthy HLA-A*02-positive donors was incubated with the indicated concentrations of ImmTAC-nybr1 or anti-CD3 (Biolegend, clone UCHT-1, cat. 300414) and anti-CD28 (BD Biosciences, clone CD28.2, cat. 555725) at 5 μg/ml. Cytokine release (IL-6, TNF-α, IL-2 and IFNγ) was measured using the Meso Scale Discovery (MSD) immunoassay.

### Alanine scanning mutagenesis

Alanine-scanning mutagenesis (ALA-scan) was performed for ImmTAC-nyeso molecule recognising the native NY-ESO-1 peptide SLLMWITQC [26]. Variants of the NY-ESO-1 peptide SLLMWITQC, in which each amino acid position was sequentially replaced with alanine, were obtained from Peptide Protein Research Ltd, UK. Synthetic peptides were tested for cross-reactivity using IFNγ ELISpot assay. Target HLA-A*02+ T2 antigen presenting cells were pulsed with native or alanine-substituted peptides and incubated with PBMC effector cells in the presence of 0.1 nM ImmTAC-nyeso. All conditions were performed in triplicate. Alanine-substituted positions that caused a 20% or greater decrease in IFNγ release, compared to the native NY-ESO-1 peptide, were considered essential and provided a binding motif. The binding motif for ImmTAC-nyeso was XLXMWIXQX, where X is any amino acid. The ScanProsite tool (http://prosite.expasy.org/scanprosite) was used to search the UniProtKB/Swiss-Prot database for proteins which contain the motif identified above. The search was limited to human sequences.

### X-scanning mutagenesis

X-scanning mutagenesis (X-scan) was carried out using NY-ESO-1 peptide (SLLMWITQC) where each amino acid position was sequentially replaced with all 19 alternative naturally-occurring amino acids (171 peptides in total) [26]. Each peptide was presented on T2 cells and IFNγ-release was measured in the presence of PBMC effector cells and 0.1 nM ImmTAC-nyeso using the ELISpot assay, as described for ALA-scan. Essential amino acid positions were defined as greater than 20% reduction in T cell activity relative to the native peptide. The ScanProsite tool (https://prosite.expasy.org/scanprosite/) was used to search for proteins containing the tolerated residues at the indicated positions (entered as [SVTYMFAGHNQKPRWLI]-[LI]-[LVAMICQNH]-[MQVTN]-W-[ITLMS]-[TSD]-[QG]-[CLVMSTGAI]).

## Results

**Fig 1:**
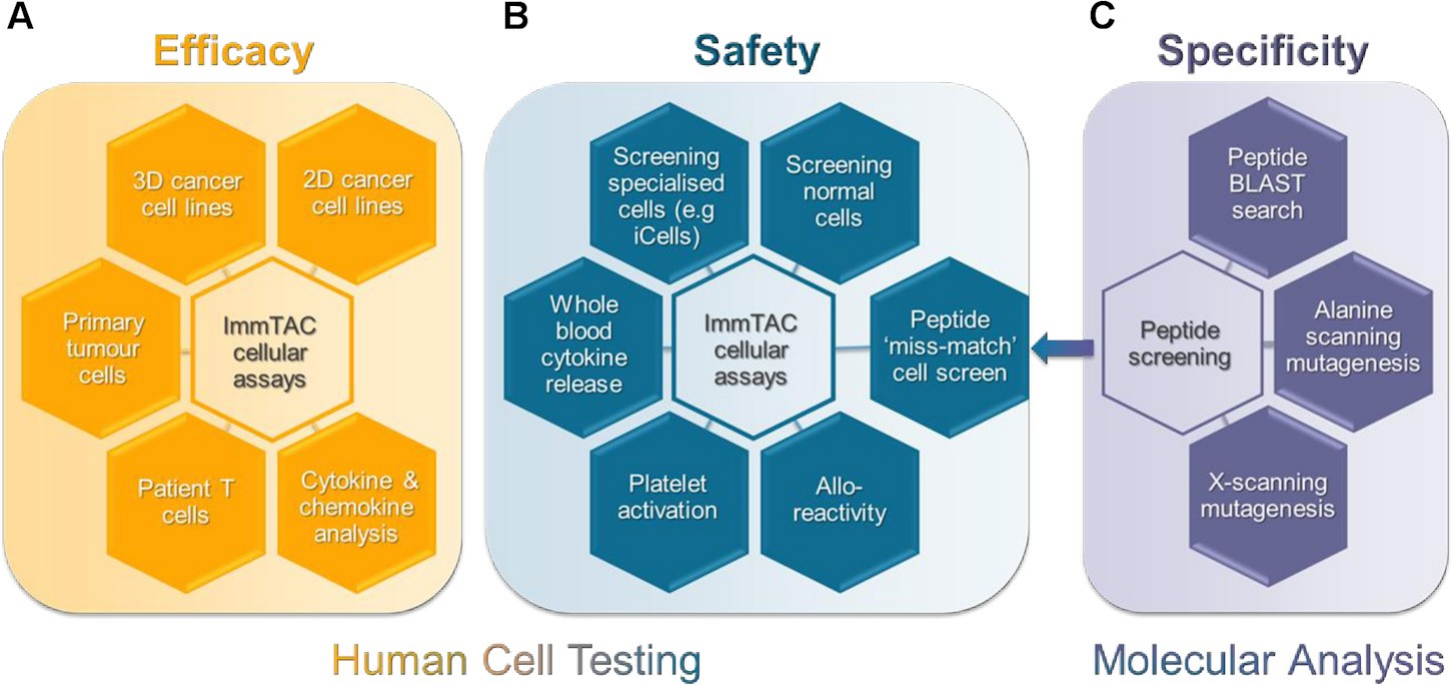
**Schematic representation of a systematic pre-clinical package to assess the (A) efficacy, (B) safety and (C) specificity of ImmTAC molecules for clinical development.** (A) The efficacy of ImmTAC molecules against a wide range of indication relevant cells presenting target peptide-HLA is assessed. These cellular assays include both patient primary tumour cells and patient T cells relevant to the indication of interest. Cytokine and chemokine analysis form a key element of efficacy measurements. (B) The safety profile of ImmTAC molecules is measured using an array of cellular assays that screen a large panel of normal and specialised antigen-positive and antigen-negative cells. ImmTAC-induced cytokine release and platelet activation is measured in whole blood. Allo-reactivity assays test cross-reactivity against different HLA-subtypes. (C) A thorough peptide screening package is used to assess potential peptide cross-reactivity, incorporating computational BLAST searches and alanine- and x-scanning peptide mutagenesis. Potential off-target peptides that are identified as closely related to the target peptide (peptide ‘missmatch’) are further screened in cellular assays to assess cross-reactivity.

Here we detail our *in vitro* preclinical testing regimen that incorporates both human cell testing and molecular analysis to assess the efficacy, safety and specificity of ImmTAC molecules (Fig 1). To assess efficacy, indication-relevant tumour cells expressing pHLA antigen (Ag^+^) target are screened using a range of sensitive T cell activation and T cell-mediated killing assays (Fig 1A). To perform safety assessments, Ag^+^/HLA-relevant normal cells are tested, as well as Ag^-^ normal cells, to determine cross-reactivity (Fig 1B). The potential of a TCR to recognise peptide presented by an alternative HLA-type is assessed using alloreactivity assays. Furthermore, systemic cross-reactivity in the blood, by either broad immune-cell activation (e.g. cytokine release syndrome) or effects on other key blood components such as platelets, is evaluated (Fig 1B). Stringent molecular analyses enriches cellular testing by predicting cross-reactive peptides through sequence analysis of the short, linear peptide in combination with *in silico* prediction. Any identified, potentially cross reactive peptides are tested in further safety screening (Fig 1C).

Data generated from the preclinical assessment of four ImmTAC molecules that progressed to different stages of development are presented. ImmTAC-gp100, ImmTAC-nybr1, ImmTAC-mageA3 and ImmTAC-nyeso, binding to gp100-HLA-A*02, NYBR1-HLA-A*02, MAGE-A3-HLA-A*01 and NY-ESO-1-HLA-A*02 complexes, respectively [28, 30]. ImmTAC-gp100 advanced into clinical trials, while ImmTAC-nybr1, ImmTAC-mageA3 and ImmTAC-nyeso molecules did not progress to investigational new drug (IND) submission. Different aspects of the preclinical evaluation of these molecules detailed in Fig 1 are discussed, along with their potential clinical implications and predictive nature.

### Cellular assessment of efficacy

#### T cell activation

**Fig 2:**
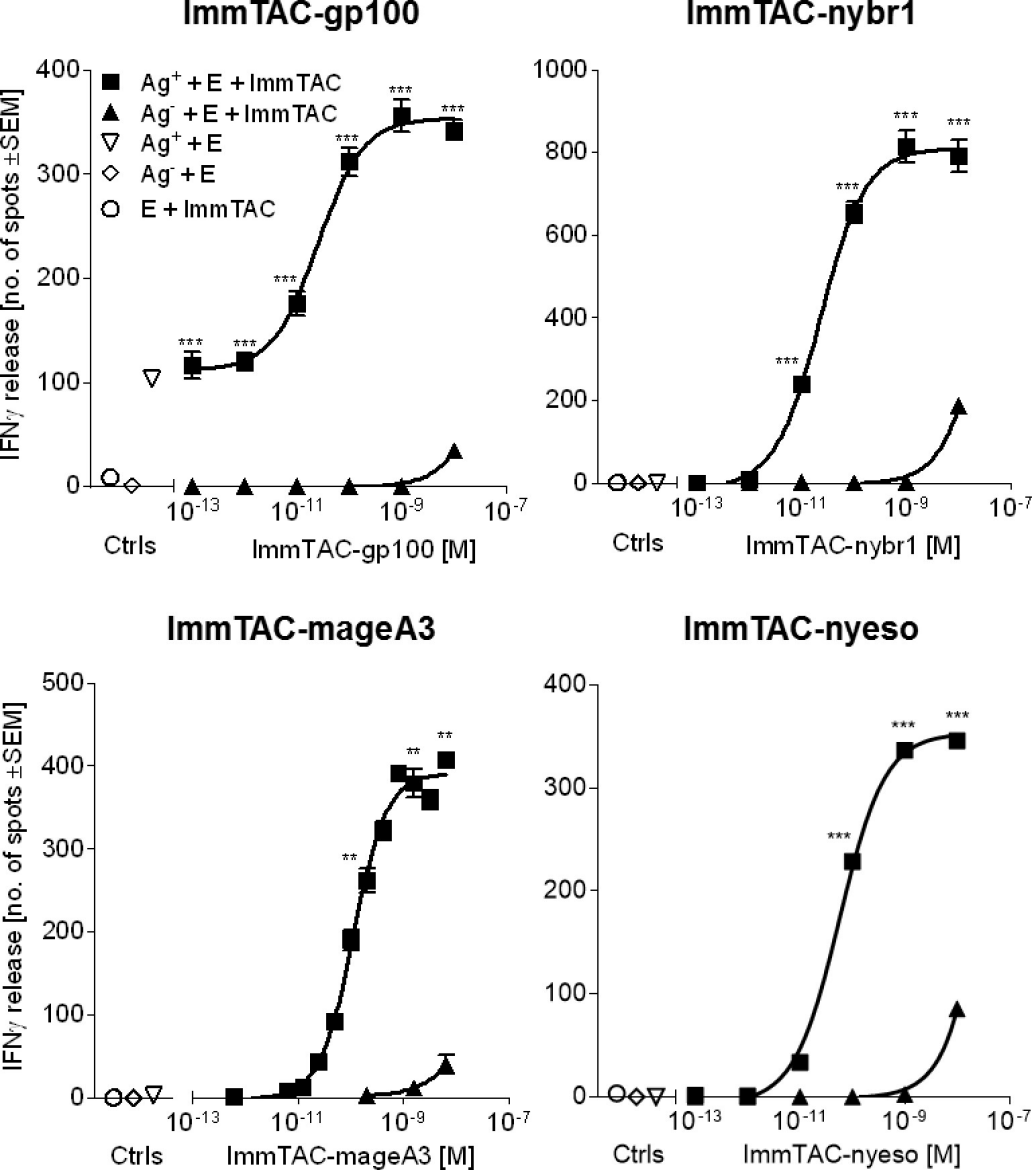
ImmTAC-mediated T cell activation and cytotoxicity. Effector cells (E = PBMCs) obtained from a melanoma patient (for ImmTAC-gp100) or healthy donors (for ImmTAC-nybr1, ImmTAC-mageA3 and ImmTAC-nyeso) were incubated with Ag+ cell lines that present target peptide-HLA or Ag- cell lines that are HLA-relevant but do not present target peptide. Cells were incubated in the presence or absence of ImmTAC molecules. IFNγ release was assessed by ELISpot assay. Ag^+^ cells: Mel526, CAMA1 A2b2m, EJM and IM9 for ImmTAC-gp100, ImmTAC-nybr1, ImmTAC-mageA3 and ImmTAC-nyeso respectively. Ag^-^ cells: A375, MDA MB 231, Colo205 and Mel526 for ImmTAC-gp100, ImmTAC-nybr1, ImmTAC-mageA3 and ImmTAC-nyeso, respectively. Statistical differences between Ag+ and Ag- cells in the presence of effector cells (E) + ImmTAC was measured using a Two-way ANOVA where *** p<0.0001, **p<0.01. If unmarked, results were not significant.

The correlation between target cell killing and redirected activation of T cells following incubation with an ImmTAC molecule is an important aspect of efficacy testing. IFNγ release was used as a measure of redirected T cell activation (using both healthy and cancer patient T cells) against Ag^+^ cell lines in the presence of antigen-specific ImmTAC molecules (Fig 2).

ImmTAC-mediated IFNγ-secretion was detected at concentrations of 1 pM, ImmTAC-gp100; 1 pM, ImmTAC-nybr1; 10 pM, ImmTAC-mageA3 and 10 pM, ImmTAC-nyeso. Maximal response was determined as 1 nM for all four ImmTAC molecules. Incubation with a high ImmTAC concentration (1 nM -10 nM) resulted in activation of T cells in the presence of cell lines devoid of the antigen, indicating a non-specific response; however, minimal levels of IFNγ were detected at this high ImmTAC concentration (Fig 2). Both healthy and cancer patients’ PBMCs enabled effective ImmTAC-mediated responses.

#### Epitope presentation and cytotoxicity

**Fig 3:**
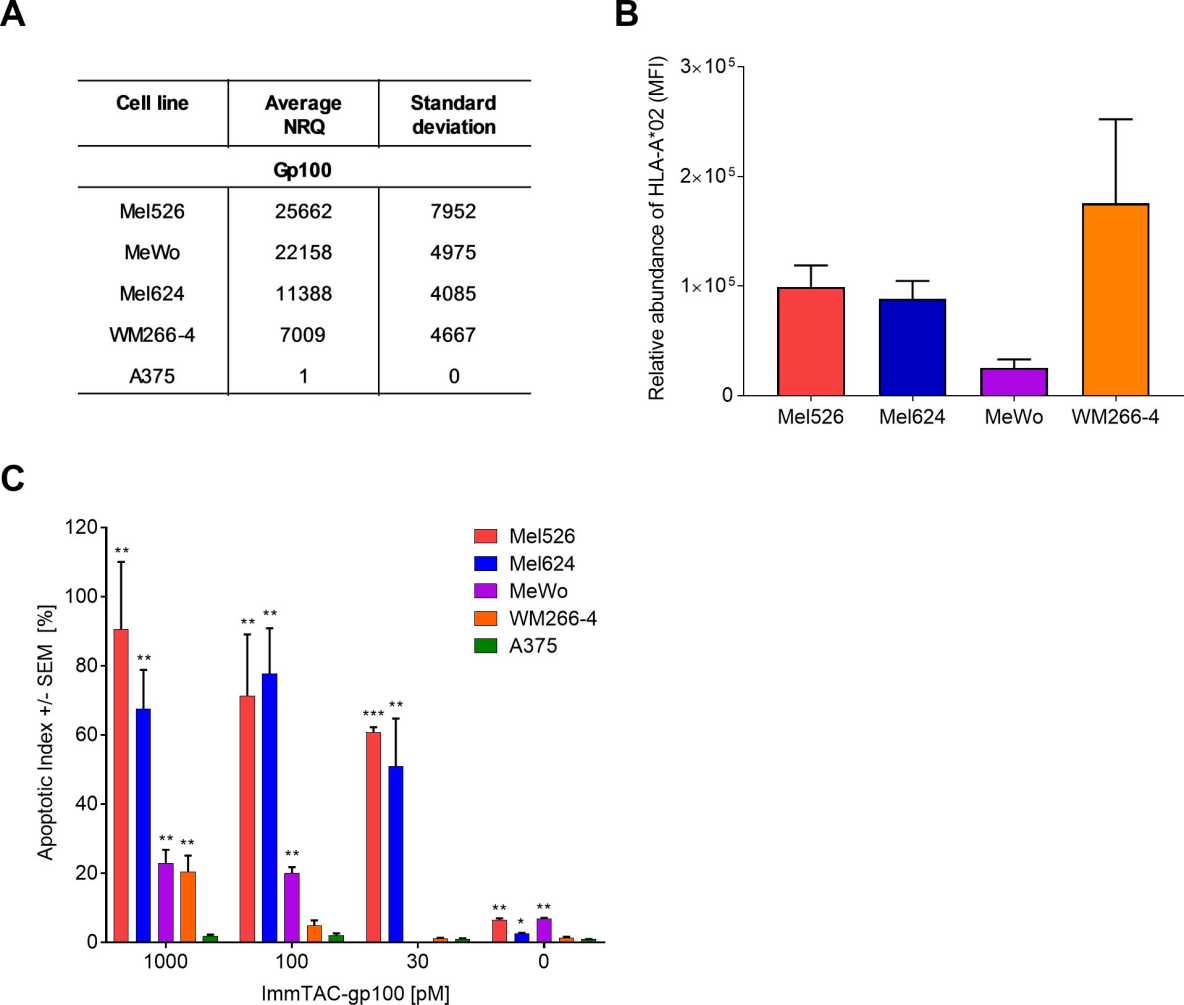
ImmTAC-mediated killing capacity is dependent on the levels of target antigen presentation. (**A**) gp100 mRNA levels measured in specified cell lines using qRT-PCR. Expression is presented as Normalised Relative Quantity (NRQ) relative to housekeeping genes (RPL32 and HPRT1). NRQ = (RQ target gene/geometric mean RQ housekeeping genes) x 10^4^, RQ = Efficiency-CT. n = 3, 4, 5, 3 and 2 for Mel526, Mel624, MeWo, WM266-4 and A375, respectively. (**B**) Levels of HLA-A*02 protein on the cell surface of specified cell lines measured by flow cytometry (mean fluorescence intensity (MFI) adjusted to the isotype control). n = 8, 9, 4 and 3 for Mel526, Mel624, MeWo, VM266-4 and A375, respectively. (**C**) Killing capacity of ImmTAC-gp100-redirected T cells assessed using the IncuCyte assay. A range of cell lines expressing different levels of gp100-HLA-A*02 complexes were incubated with decreasing concentrations of ImmTAC-gp100. The apoptotic index was determined by calculating the % ratio of apoptotic cells to the total number of target melanoma cells at the experimental endpoint (52 hours). n = 2–3. Statistical differences in apoptotic index between Ag+ (Mel 526, Mel624, MeWo and WM266-4) and Ag- (A375) cell lines were individually measured at each concentration of ImmTAC molecule using an unpaired T-test where *** p<0.0001, **p<0.01 and *p<0.05. If unmarked, results were not significant.

Another aspect of ImmTAC efficacy testing is examining the level of target antigen presented by the indication-relevant tumour cells. Here, we demonstrate the correlation between transcript level, HLA-surface expression and target cell killing (Fig 3).

ImmTAC-gp100 mediated dose-dependent T cell-driven apoptosis of gp100-positive target cells, while gp100-negative cells (A375) were not targeted (Fig 3C). High levels of T cell-driven apoptosis observed for Mel526 and Mel624 cells correlated with high gp100 mRNA levels and HLA-A*02 protein presentation. Comparable levels of apoptosis between Mel526 and Mel624 cells is supported by our previously published data that showed comparable levels of gp100 peptide epitopes on the cell surface (~37 and ~37, respectively) [31]. MeWo cells also showed an abundance of gp100 transcripts (Fig 3A), though HLA-A*02 protein levels were low (Fig 3B), likely accounting for their relatively moderate levels of cell death (Fig 3C). Conversely, WM266-4 cells showed high HLA-A*02 protein levels (Fig 3B) but low gp100 mRNA levels (Fig 3A) translating to lower levels of T-cell driven apoptosis (Fig 3C).

Together these data demonstrate that peptide expression is dictated by both cellular mRNA levels and HLA-A*02 protein expression, which together inform on the efficacy of a given ImmTAC molecule.

#### In vitro MABEL

Calculation of MABEL is used as a conservative approach to derive a safe starting dose for clinical testing, drawing on multiple assays to identify the most sensitive measurement of efficacy. To determine a safe clinical starting dose for ImmTAC-gp100, data from IFNγ-release, epitope presentation and killing capacity using lactate dehydrogenase (LDH) release as a sensitive kinetic measure of cell cytotoxicity, were assessed [32].

LDH release data for ImmTAC-gp100 correlated with both the efficiency of killing in the IncuCyte assay (S1 Fig and Fig 3C) and redirected T cell activation, measured by IFNγ release (Fig 2). Maximum killing of the target cells and the peak of specific cell lysis were achieved at around the same concentration of ImmTAC-gp100 across assays (0.1 nM—1 nM).

These data contributed to the determination of MABEL for ImmTAC-gp100 as 1 pM, the lowest tested concentration of ImmTAC-gp100 that caused a detectable biological effect in an assay, in this case, an increase in IFNγ- and LDH-release over the baseline (Fig 2 and S1 Fig). Above 1 nM ImmTAC-gp100, cytolysis of gp100-negative cells (A375) was detected (Fig 2), albeit markedly lower than gp100-positive cells. This non-specific response determined the upper dose threshold as 1nM.

### Cellular assessment of safety and specificity

#### On-target, off-tumour activity

**Fig 4:**
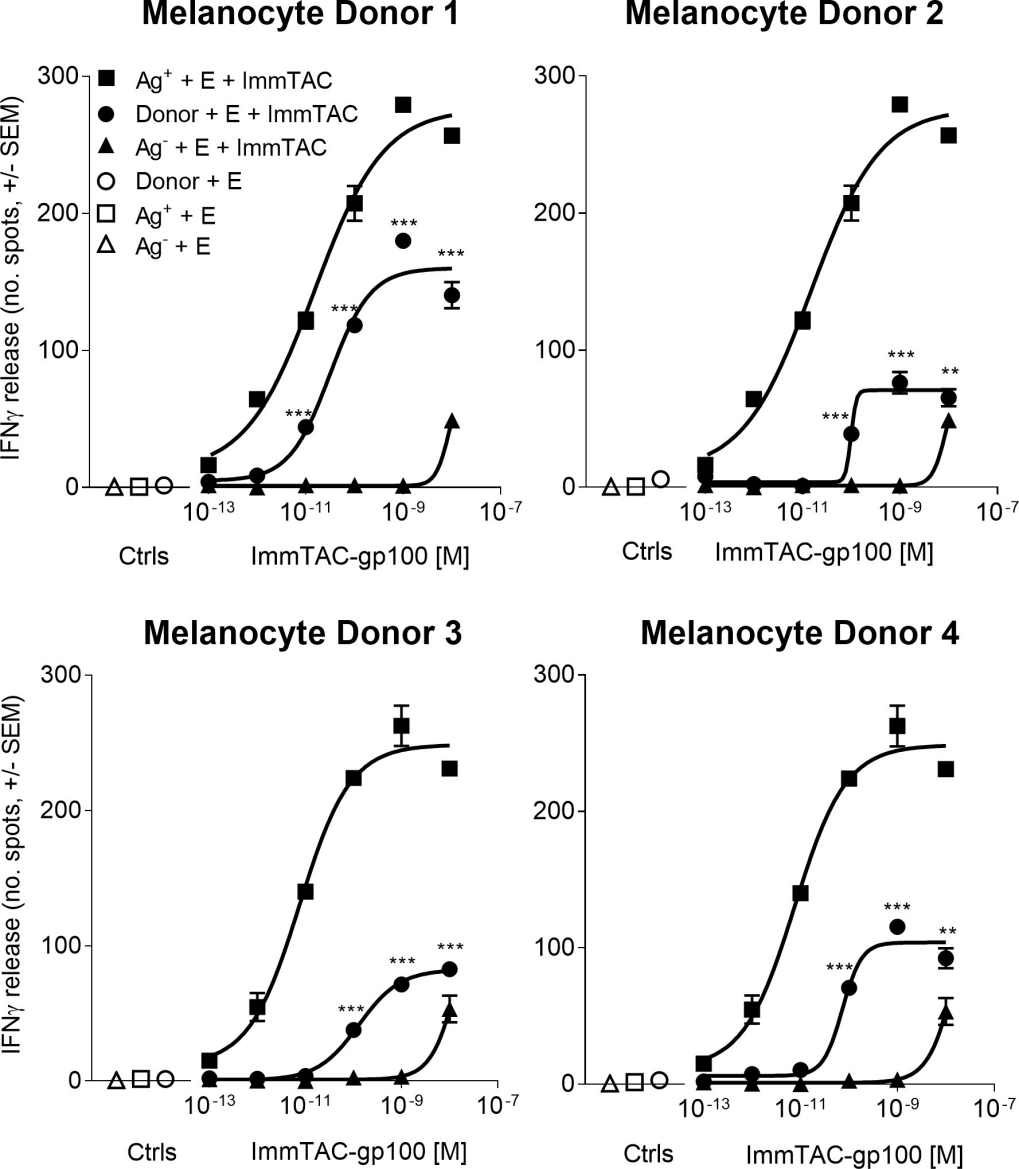
On-target, off-tumour activity of ImmTAC-gp100 against skin melanocytes. IFNγ release was measured by ELISpot assay from normal human epidermal melanocytes (NHEMs) from four HLA-A*02-positive healthy donors (Melanocyte Donors 1–4), gp100 +ve (Ag+) melanoma cells (Mel526 cell line) and gp100 –ve (Ag-) control melanoma cells (A375 cell line) incubated with polyclonal (non-tumour specific) PBMCs effector cells (E) in the presence or absence of increasing concentrations of gp100-specific ImmTAC molecule (ImmTAC-gp100). Results presented represent the most reactive PBMC donor tested. Statistical differences in IFN γ release between donor NHEM cells and Ag- melanoma cells in the presence of effector cells (E) + ImmTAC molecule was measured using a Two-way ANOVA where *** p<0.0001, **p<0.01. If marked, results were not significant.

The assessment of on-target, off-tumour effects requires a thorough understanding of the target antigen expression profile, which for gp100 is not confined to tumour cells, but is also present in healthy melanocytes. To better understand and predict the nature for potential toxicity in the clinic, ImmTAC-gp100-dependent activation of redirected T cells was assessed by measuring IFNγ-release in the presence of melanocytes derived from gp100-positive, HLA-A*02-positive donors, alongside a reference melanoma cell line (Mel526) (Fig 4).

The most reactive PBMC donor is presented and was considered a guideline for the potential on-target, off-tumour activity of ImmTAC-gp100 in patients (Fig 4). Responses for all melanocyte donors were ImmTAC-gp100 dose-dependent (0.1 pM to 10 nM) and consistently lower than for Mel526 Ag^+^ cells. No T cell mediated response was observed for melanocytes or melanoma cells when incubated in the absence of ImmTAC. Loss of ImmTAC specificity was observed between 1 nM and 10 nM, detected by IFNγ-release from the A375 antigen-negative cell line.

Three melanocyte donors (NHEM2-4) induced an initial T cell response between 10 pM and 100 pM, whilst melanocytes from the most sensitive donor (NMEM1) triggered IFNγ release between 1 pM and 10 pM of ImmTAC-gp100. These data suggest that adverse events related to skin could be expected from 1 pM -10 pM in the most sensitive donors and in the 10 pM -100 pM range for the majority of patients treated with ImmTAC-gp100.

Based on MABEL, determined as 1 pM, and the loss of ImmTAC specificity at 1 nM, the therapeutic window as defined by these observations is predicted to be sufficiently wide to treat patients with an expansion clinical dose in the range of 10 pM– 100 pM. Given the potential benefits of ImmTAC-gp100 therapy, a degree of on-target off-tumour activity against skin melanocytes was deemed an acceptable risk, particularly with the incorporation of appropriate safety management in a clinical trial setting.

#### Assessing off-target, off-tumour activity

**Fig 5:**
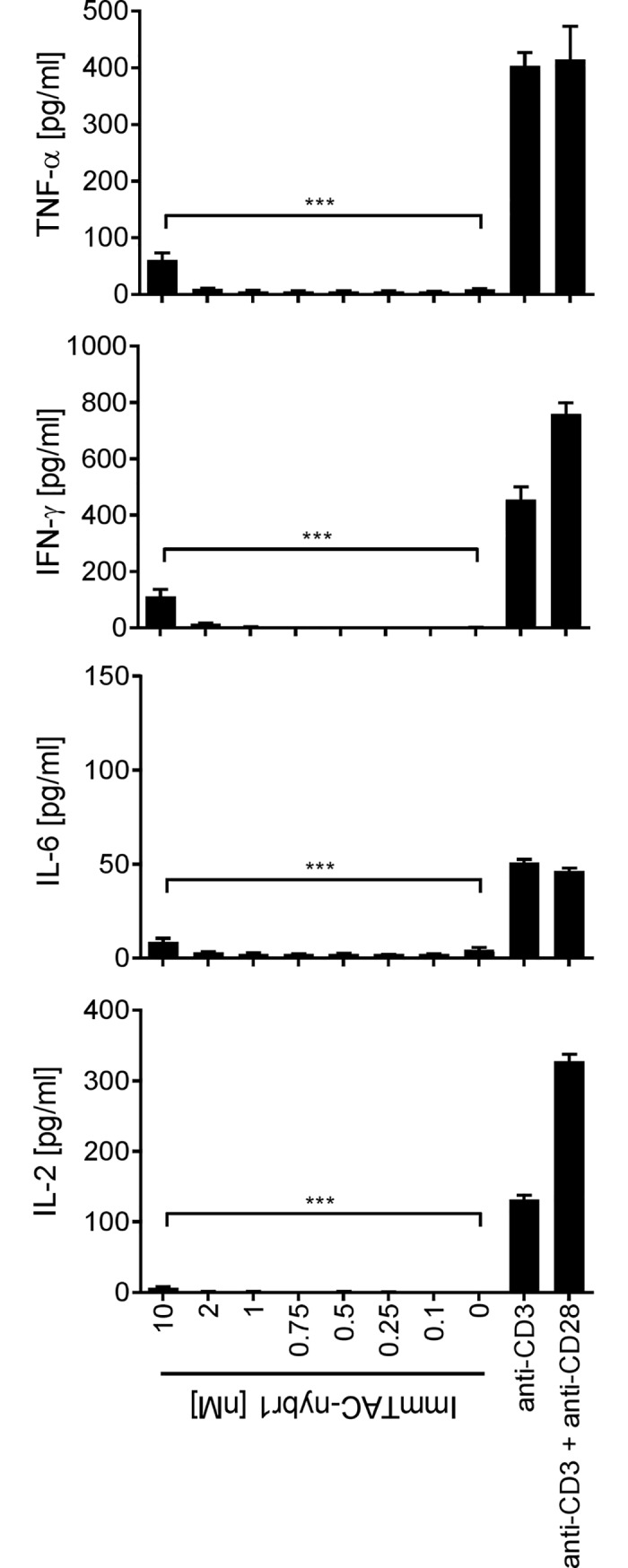
Cellular safety and specificity of ImmTAC-nybr1 in whole blood. Whole blood freshly isolated from four healthy HLA-A*02-positive donors was incubated with increasing concentrations (0.1 nM -10 nM) of NYBR1-specific ImmTAC molecule (ImmTAC-nybr1) and the release of cytokines (IL-2, IL-6, IFNγ and TNFα) was measured using the Meso Scale Discovery (MSD) assay. Whole blood incubated with an anti-CD3 antibody alone or in combination with anti-CD28 antibody were used as positive controls. Representative results from one donor are presented. Statistical differences in cytokine release in the presence or absence of ImmTAC molecule at each concentration tested (0.1 nM—10 nM) were measured using a One-way ANOVA where *** p<0.0001. If unmarked, results were not significant.

The most likely source of off target effects is recognition of an alternative, yet similar peptide, complexed to the compatible or different HLA-type. As part of this safety assessment, the potential for ImmTAC molecules to elicit broad immune activation is determined by measuring cytokine release in whole blood. Cytokine release data in whole blood is shown for the example ImmTAC molecule ImmTAC-nybr1 (Fig 5). ImmTAC-nybr1, at a concentration of 2 nM or lower, did not trigger the release of cytokines IL-2, IL-6, IFNγ or TNFα above the levels observed in the absence of ImmTAC-nybr1 (Fig 5). Low levels of cytokine release were observed at high concentrations of ImmTAC-nybr1 (10 nM), a concentration ten-fold above that required to deliver maximal T cell activation (Fig 2).

**Fig 6:**
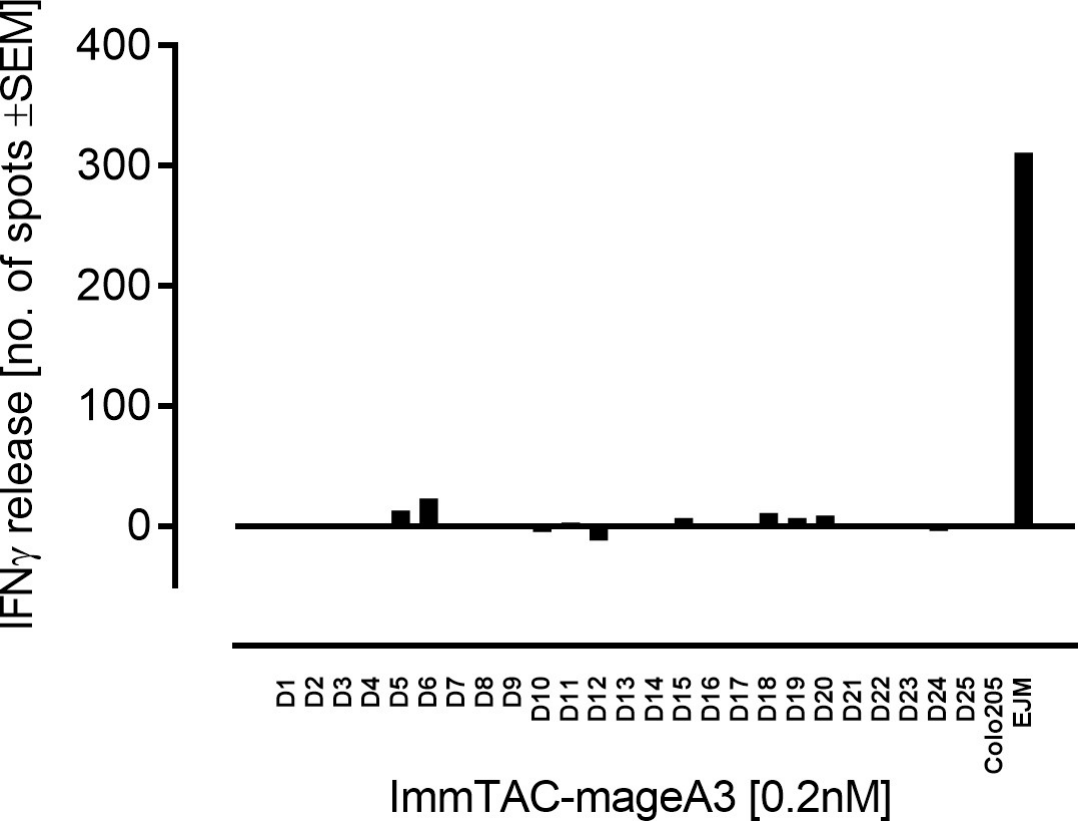
Alloreactivity assessment of ImmTAC molecules. Alloreactivity potential of ImmTAC-mageA3 recognising MAGE-A3 peptide in the context of HLA-A*01 was measured across cells from donors (D1-25) bearing a range of HLA types using the IFNγ ELISpot assay. Donor cells were isolated and incubated with polyclonal CD8+ T cells in the presence or absence of 0.2 nM ImmTAC-mageA3. IFNγ-release recorded in the absence of ImmTAC-mageA3 was subtracted from measurements (n = 3) and plotted as means. EJM (Ag^+^ & HLA-A*01^+^) and Colo205 (Ag^-^ & HLA-A*01^+^) cell lines were used as controls. No statistical differences in IFNγ-release were observed across donor cell types when compared to IFNγ-release in the absence of ImmTAC molecule, measured using paired, one-tailed t-tests.

To determine the potential of an ImmTAC molecule to recognise non-target HLA subtypes, ImmTAC-mageA3 recognising MAGE-A3 peptide in the context of HLA-A*01 was used as an example ImmTAC molecule to assess alloreactivity potential of redirected T cells against a broad panel of cells/cell lines, each of a unique HLA-type. The comprehensive panel covers all HLA-types that exist at frequencies of >10% of the target population. ImmTAC-mageA3 caused no significant IFNγ-release across the panel of donor cells tested (Fig 6).

### Molecular assessment of safety and specificity

As a minimal approach to molecular analysis of TCR peptide recognition, *in silico* analysis can be performed (for example using BLAST program) to identify peptides within the human genome that share a high percentage of sequence identity to the target. However, *in silico* analysis is not always sufficient, as exemplified by the MAGE-A3 TCR designed for adoptive cell therapy that caused fatal toxicity in two patients due to cross-reactivity against a peptide mimic from the titin protein presented on cardiac tissue [28, 33].

**Fig 7:**
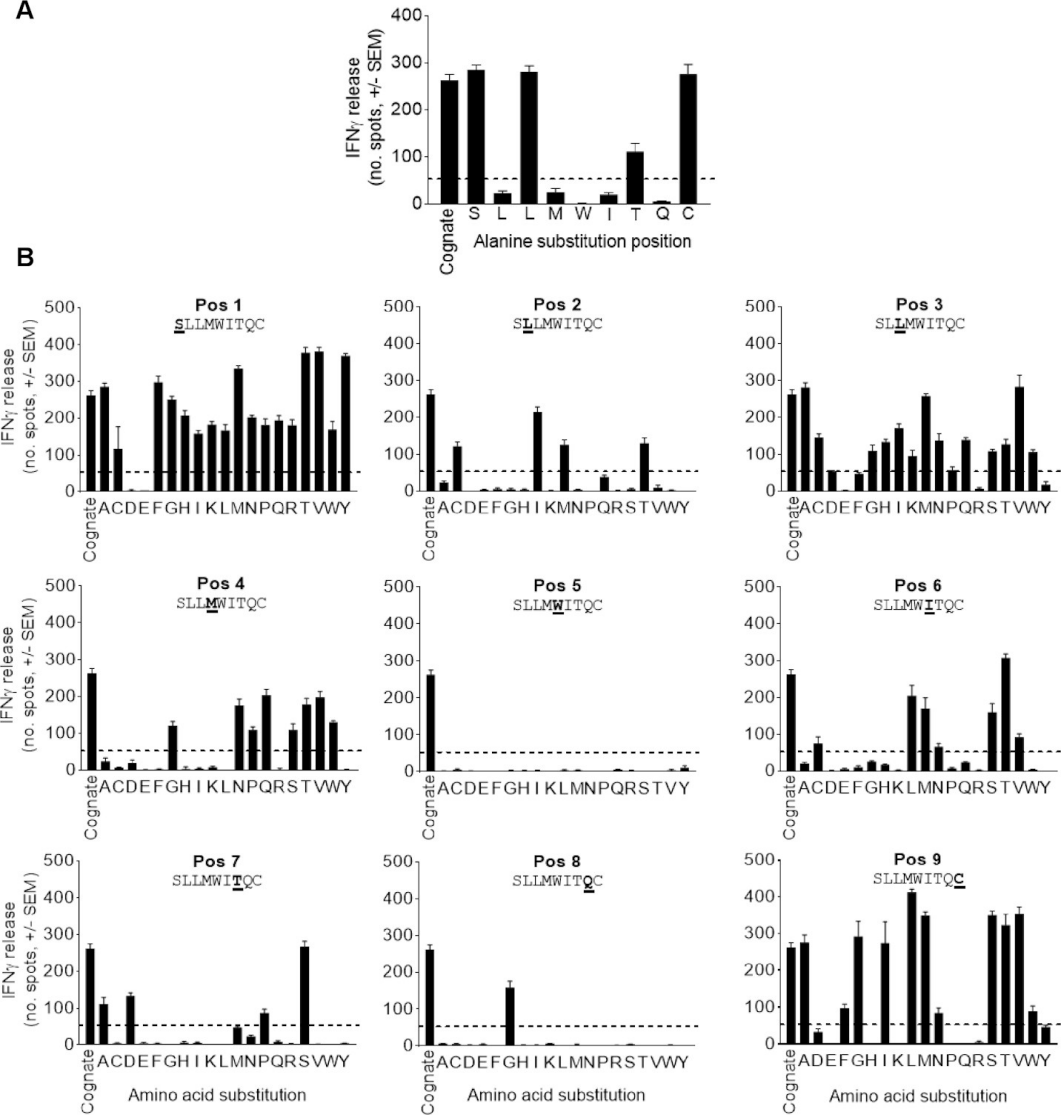
Molecular analysis of ImmTAC specificity for NY-ESO-1 peptide (SLLMWITQC). (**A**) Each amino acid in the target peptide sequence was individually replaced by alanine (alanine-scanning mutagenesis) and each of the 9 single-mutated peptides were pulsed onto T2 HLA-A*02^+^ cells. Pulsed T2 cells presenting peptide were incubated with PBMCs from three healthy donors with 0.1 nM ImmTAC-nyeso molecule and IFNγ-release was measured by ELISpot assay. Representative results from one donor are presented. Dotted lines indicate 20% of cognate peptide reactivity (**B**) Each amino acid in the peptide sequence was exchanged by each of the 19 amino acids (X-scanning mutagenesis). Activation of PBMCs by peptide pulsed T2 cells in the presence of 0.1 nM ImmTAC-nyeso was measured by IFNγ ELISpot assay.

More in depth molecular assessment can be performed using ALA-scan; replacing each residue in the target peptide with alanine and testing the potential of each mutated peptide presented on a T2 antigen presenting cell to cause T cell activation. An example of an ALA-scan is presented for NY-ESO-1 peptide (Fig 7A). ImmTAC-nyeso recognised peptides with a mutated amino acid at positions: 1, 3, 7 and 9 as indicated by IFNγ release levels of at least 20% of the cognate peptide, highlighting these positions as potentially cross-reactive.

More comprehensive TCR motif assessment is achieved using an X-scan, substituting each amino acid of the target peptide to one of the 19 remaining amino acids (Fig 7B) [26]. Positions identified as restrictive from ALA-scan were not always restrictive in the more extensive X-scan. For example, substitution of Ile at position 6 in NY-ESO-1 peptide with Cys, Leu, Met, Asn, Ser, Thr or Val resulted in ImmTAC-nyeso recognition and IFNγ release.

Combined information from ALA-scan and X-scan allows a motif to be determined that is subject to *in silico* analysis, cross-referencing against an internal database containing mass spectrometry-identified peptides presented by an extensive range of human cell lines. Any peptides from BLAST searches that conform to the TCR binding motif and have direct evidence for presentation on HLA are considered potentially cross-reactive and subject to further cell testing. Based partly on the learnings from the fatally cross-reactive MAGE-A3 TCR [28], cellular testing of ImmTAC molecules has extended to include specialised human cell cultures, including induced pluripotent stem cells (iPSC), to capture an even wider scope of the human peptidome.

## Discussion

ImmTAC molecules are entirely human-specific, targeting human pHLA complexes with an anti-CD3 effector function that specifically activates human T cells, thus conventional preclinical animal models are inappropriate to support clinical studies. Here, we detail a novel, validated approach to preclinical testing that can also be applied to other TCR-based therapeutics to facilitate calculation of safe clinical starting dose, predict potential clinical toxicities and inform on appropriate clinical trial design. Our entirely *in vitro* preclinical package is exemplified by drawing upon experience from preclinical testing of multiple example ImmTAC molecules, including ImmTAC-gp100 (referred to in the clinic as IMCgp100) that advanced into clinical trials.

Applying ImmTAC-gp100 as an example ImmTAC molecule for efficacy assessment, we describe a clear correlation between ImmTAC potency and target antigen (pHLA) presentation, which is further dependant on the abundance of the target mRNA transcript levels and expression of the appropriate HLA subtype. We previously published that fewer than 20 epitopes displayed on the surface of a cell are sufficient to trigger potent ImmTAC-driven apoptosis [6, 34]. Thus ImmTAC molecules are able to overcome the limitations of natural TCRs and target immune-evading cancer cells that often present low levels of pHLA. The ability to effectively target low level pHLA antigens maximises the therapeutic potential of ImmTAC molecules, enabling access to a substantially broader repertoire of antigenic targets than is available to traditional antibody-based therapies.

In this study, the two most sensitive measurements of efficacy studied; target cell killing by cell lysis or cell apoptosis, were used as part of an example method to anticipate safe starting dose for first-in-human trials. Using the most sensitive assay, a predictive MABEL for ImmTAC-gp100 could be determined (1pM). Notably, effector T cells from healthy donors and cancer patients mounted comparable immune responses against target cells indicating that cancer patients’ immune systems were not compromised.

Based on the efficacy assessment observations, the therapeutic window for ImmTAC-gp100 was predicted to allow dose expansion in the range of 10 pM to 100 pM. With this in mind, the dose-dependent on-target off-tumour reactivity of ImmTAC-gp100 against skin melanocytes, know to present gp100 peptide, was investigated using primary melanocyte cultures to further inform on the potential therapeutic window for ImmTAC-gp100 in the clinic. It was anticipated that skin toxicity may be clinically observed in the most sensitive patients from 1 pM to 10 pM of ImmTAC-gp100. Given the relative low abundance of melanocytes in the skin (up to 5% of the cells in epidermis) [35] and the relative tolerance to toxicity in this organ, it is anticipated that a proinflammatory response in this compartment would be tolerated and managed in the clinic.

The potential of immune-modulating therapies to elicit off-target off-tumour adverse systemic activity is a key safety consideration. There are accumulating examples where immuno-therapeutic agents have resulted in severe adverse events and fatal toxicities, often resulting from systemic cytokine release syndrome, that were not sufficiently predicted from preclinical evaluation. Among the reported adverse incidences of cytokine release syndrome are the severe toxicities observed in multiple healthy volunteers in the immune-activating TGN1412 anti-CD28 monoclonal antibody trial, incidences of neurotoxicity in patients treated with the anti-CD19/anti-CD3 monoclonal antibody bispecific blinatumomab, as well as reports of neurotoxicity and fatalities in patients across adoptive cell transfer of CD19 chimeric antigen receptor (CAR) trials [15, 36, 37]. To mitigate the risk of systemic cytokine storm, whole blood *in vitro* assays exist in multiple formats as an essential, widely used and validated component of preclinical safety testing [38, 39]. In this study, the release of key proinflammatory cytokines, IL-6, TNFα, IL-2, IFNγ, were assessed in response to ImmTAC administration. ImmTAC-nybr1 demonstrated only minimal cytokine release at the highest ImmTAC concentration tested, a concentration ten-fold above that required to deliver maximal T cell activation.

A final element of preclinical testing concerns safety and specificity screening that assesses the potential for an ImmTAC molecule to cross react with different HLA subtypes and/or peptide ‘mimics’. Although ImmTAC molecules are specifically engineered to recognise the target peptide complexed to a specific HLA subtype, extensive measures are taken to ensure that the risk of cross-reactivity is minimised. A comprehensive panel of HLA subtypes are meticulously selected based on the target patient population and screened against ImmTAC molecules. To better interrogate the potential for cross-reactivity against ‘mimic’ peptides, ALA-scan and X-scan assays used introduced to define a TCR-binding motif, allowing potential cross-reactivitiy to be assessed in the most stringent manner. Unlike antibody-antigen recognition, ImmTAC molecules recognise a short linear peptide antigen sequence to which a complete binding motif can be generated and the entire landscape of potential mimetic peptides can be defined and empirically tested. When searched against the known human proteome, the TCR binding motif can identify potentially cross-reactive peptides. Whilst this remains a hypothetical risk (as only a fraction of any given amino acid sequence will be processed and presented on class I HLA), various additional tools are used to mitigate and/or understand this potential risk further. By searching an in-house mass spectrometry database for the existence of any hypothetical peptide sequence identified through either the ALA- or X-scans, we can determine if there is evidence for processing and presentation of that peptide sequence. Peptides with evidence of presentation can be further tested in the relevant cellular assays.

An alternative approach to cross-reactivity testing is the application of tissue immunohistochemistry (IHC) for which regulatory bodies such as the FDA define key lists of normal tissues (regulatory body-dependent) in which biologics are recommended to be screened against. However, a key challenge when applying high affinity TCRs as a probe is the low abundance (10s-100s copies per cell) of target peptide that is insufficient for IHC detection and sensitivity [6]. Although ImmTAC molecules are routinely screened using tissue cross-reactivity panels, ImmTAC-mageA3 failed to react with either testis (where the target antigen is expressed) or cardiac and muscle tissues (where the known off-target titin peptide is expressed), while cross-reactivity of ImmTAC-mageA3 could be determined using ALA-scan methodology [27]. These findings support the hypothesis that the *in vitro* functional tests described here are more sensitive and reliable than tissue IHC assays in assessing safety of TCR-based therapeutics, with currently available methods.

The molecular component of the *in vitro* package is designed to be a comprehensive representation of the peptidome existing in different contexts. Thus, an important extension to the cellular safety assays moving forward is the inclusion of more specialised cell types within the normal cell panels that add to the rigor of the *in vitro* safety package and help predict toxicities that may occur *in vivo*. For example, induced pluripotent stem cells (iPSC) and 3D cultures can provide additional coverage and representation of specific *in vivo* cellular expression patterns that might be lost in normal cultured cells. For example, in the case of the adoptively transferred affinity enhanced engineered TCR targeting MAGE-A3, which showed fatal cross-reactivity against the cardiac peptide derived from the titin protein in humans; no concerns were identified in normal cardiac myocytes, only against cardiac iCells [27, 28].

Although iPSCs create a potentially more physiologically relevant system that may significantly differ or drift in protein expression from normal cell cultures, the caveat of these models is that the cells differentiated from human pluripotent stem cells often differ from their adult counterparts. For example, cardiomyocytes derived from stem cells do not only display functional features of foetal cells but also present a gene expression profile similar to that of a first trimester foetal heart [40]. This means that iPSC cultures may present pHLA complexes that are unlikely to be found on the surface of adult cells and therefore irrelevant in terms of safety and specificity screening of a therapeutic. Consequently, iPSC models should complement the standard normal cultures rather than replace them and results from these studies need to be carefully investigated and confirmed.

Our preclinical package overcomes the species limitations of traditional preclinical testing by using human tissues, cells and *in vitro* models. A similar *in vitro* strategy to investigate cross-reactivity of biologics is being developed by others such as Retrogenix, whose human cell microarray technology for identifying off-target effects has been successfully applied to test antibodies, bispecifics, whole CAR-T cells, peptides or labelled small molecules [41]. Many of the emerging immunotherapeutics, whose biological properties cannot be fully assessed by *in vivo* testing with conventional animal toxicology models, will require testing with human tissues and cells as described here. We believe that our preclinical package can be adapted for efficacy/safety screening of other human-specific biologics, and in particular for TCR-based therapies.

## Supporting information

S1 Table Cell lines used in this study. R10; 10% RPMI, 1% FCS, 1% Penicillin/Streptomycin and 1% Glutamine. EMEM; Eagle’s Minimum Essential Medium. DMEM; Dulbecco’s Modified Eagles Medium. IMDM; Iscove’s Modified Dulbecco’s Media. MGM M2; serum-free, PMA-free optimal Melanocyte Growth Media. MGM-4; Melanocyte Growth Medium-4.
(TIF)

S1 Fig ImmTAC-mediated cytotoxicity measured Lactate Dehydrogenase (LDH) release. Ag+ cell lines (Mel526) that present target peptide-HLA and Ag- cell lines (A375) that do not present peptide-HLA were incubated with PBMC effector cells (E) from healthy donors in the presence or absence of ImmTAC-gp100 at increasing concentrations and LDH release was measured. Statistical difference between Ag+ and Ag- cells in the presence of effector cells (E) + ImmTAC was measured using a Two-way ANOVA with Sidak’s multiple comparison test where *** p<0.0001, **p<0.01.
(TIF)

S1 Dataset Raw Data.xlsx. (XLSX)

## References

[1] Farkona S, Diamandis EP, Blasutig IM. Cancer immunotherapy: the beginning of the end of cancer? BMC medicine. 2016;14:73 10.1186/s12916-016-0623-5 27151159 PMC4858828

[2] Sharma P, Wagner K, Wolchok JD, Allison JP. Novel cancer immunotherapy agents with survival benefit: recent successes and next steps. Nature reviews Cancer. 2011;11(11):805–12. 10.1038/nrc3153 22020206 PMC3426440

[3] Pardoll DM. The blockade of immune checkpoints in cancer immunotherapy. Nature reviews Cancer. 2012;12(4):252–64. 10.1038/nrc3239 22437870 PMC4856023

[4] Hodi FS, O'Day SJ, McDermott DF, Weber RW, Sosman JA, Haanen JB, et al Improved survival with ipilimumab in patients with metastatic melanoma. The New England journal of medicine. 2010;363(8):711–23. 10.1056/NEJMoa1003466 20525992 PMC3549297

[5] Rosenberg SA, Restifo NP, Yang JC, Morgan RA, Dudley ME. Adoptive cell transfer: a clinical path to effective cancer immunotherapy. Nature reviews Cancer. 2008;8(4):299–308. 10.1038/nrc2355 18354418 PMC2553205

[6] Liddy N, Bossi G, Adams KJ, Lissina A, Mahon TM, Hassan NJ, et al Monoclonal TCR-redirected tumor cell killing. Nat Med. 2012;8:980–7. 10.1038/nm.2764 22561687

[7] Morgan RA, Chinnasamy N, Abate-Daga D, Gros A, Robbins PF, Zheng Z, et al Cancer regression and neurological toxicity following anti-MAGE-A3 TCR gene therapy. Journal of immunotherapy. 2013;36(2):133–51. 10.1097/CJI.0b013e3182829903 23377668 PMC3581823

[8] Morgan RA, Yang JC, Kitano M, Dudley ME, Laurencot CM, Rosenberg SA. Case report of a serious adverse event following the administration of T cells transduced with a chimeric antigen receptor recognizing ERBB2. Molecular therapy: the journal of the American Society of Gene Therapy. 2010;18(4):843–51. 20179677 10.1038/mt.2010.24 PMC2862534

[9] Lowe KL, Mackall CL, Norry E, Amado R, Jakobsen BK, Binder G. Fludarabine and neurotoxicity in engineered T-cell therapy. Gene Ther. 2018;25(3):176–91. 10.1038/s41434-018-0019-6 29789639

[10] Fox BA, Schendel DJ, Butterfield LH, Aamdal S, Allison JP, Ascierto PA, et al Defining the critical hurdles in cancer immunotherapy. Journal of translational medicine. 2011;9:214 10.1186/1479-5876-9-214 22168571 PMC3338100

[11] Mould DR, Meibohm B. Drug Development of Therapeutic Monoclonal Antibodies. BioDrugs: clinical immunotherapeutics, biopharmaceuticals and gene therapy. 2016;30(4):275–93. 10.1007/s40259-016-0181-6 27342605

[12] Pavlovic D, Patera AC, Nyberg F, Gerber M, Liu M, Progressive Multifocal Leukeoncephalopathy C. Progressive multifocal leukoencephalopathy: current treatment options and future perspectives. Therapeutic advances in neurological disorders. 2015;8(6):255–73. 10.1177/1756285615602832 26600871 PMC4643867

[13] Beyersdorf N, Gaupp S, Balbach K, Schmidt J, Toyka KV, Lin CH, et al Selective targeting of regulatory T cells with CD28 superagonists allows effective therapy of experimental autoimmune encephalomyelitis. The Journal of experimental medicine. 2005;202(3):445–55. 10.1084/jem.20051060 16061730 PMC2213080

[14] Hunig T, Dennehy K. CD28 superagonists: mode of action and therapeutic potential. Immunology letters. 2005;100(1):21–8. 10.1016/j.imlet.2005.06.012 16054703

[15] Suntharalingam G, Perry MR, Ward S, Brett SJ, Castello-Cortes A, Brunner MD, et al Cytokine storm in a phase 1 trial of the anti-CD28 monoclonal antibody TGN1412. The New England journal of medicine. 2006;355(10):1018–28. 10.1056/NEJMoa063842 16908486

[16] Ryan PC. In vitro MABEL approach for nonclinical safety assessment of MEDI-565 (MT111). Altex Proceedingss, 1/12, Proceedings of WC82012. 85–7 p.

[17] Shultz LD, Brehm MA, Garcia-Martinez JV, Greiner DL. Humanized mice for immune system investigation: progress, promise and challenges. Nature reviews Immunology. 2012;12(11):786–98. 10.1038/nri3311 23059428 PMC3749872

[18] Covassin L, Laning J, Abdi R, Langevin DL, Phillips NE, Shultz LD, et al Human peripheral blood CD4 T cell-engrafted non-obese diabetic-scid IL2rgamma(null) H2-Ab1 (tm1Gru) Tg (human leucocyte antigen D-related 4) mice: a mouse model of human allogeneic graft-versus-host disease. Clinical and experimental immunology. 2011;166(2):269–80. 10.1111/j.1365-2249.2011.04462.x 21985373 PMC3219902

[19] Sewell F, Chapman K, Couch J, Dempster M, Heidel S, Loberg L, et al Challenges and opportunities for the future of monoclonal antibody development: Improving safety assessment and reducing animal use. mAbs. 2017;9(5):742–55. 10.1080/19420862.2017.1324376 28475417 PMC5524158

[20] Brehm MA, Shultz LD, Luban J, Greiner DL. Overcoming current limitations in humanized mouse research. The Journal of infectious diseases. 2013;208 Suppl 2:S125–30. 24151318 10.1093/infdis/jit319 PMC3807974

[21] van Meer PJ, Kooijman M, Brinks V, Gispen-de Wied CC, Silva-Lima B, Moors EH, et al Immunogenicity of mAbs in non-human primates during nonclinical safety assessment. mAbs. 2013;5(5):810–6. 10.4161/mabs.25234 23924803 PMC3851233

[22] van Lent AU, Dontje W, Nagasawa M, Siamari R, Bakker AQ, Pouw SM, et al IL-7 enhances thymic human T cell development in "human immune system" Rag2-/-IL-2Rgammac-/- mice without affecting peripheral T cell homeostasis. Journal of immunology. 2009;183(12):7645–55. 10.4049/jimmunol.0902019 19923447

[23] Merlino G, Flaherty K, Acquavella N, Day CP, Aplin A, Holmen S, et al Meeting report: The future of preclinical mouse models in melanoma treatment is now. Pigment cell & melanoma research. 2013;26(4):E8–E14. 23531109 10.1111/pcmr.12099 PMC4109982

[24] McKim JM Jr. Building a tiered approach to in vitro predictive toxicity screening: a focus on assays with in vivo relevance. Combinatorial chemistry & high throughput screening. 2010;13(2):188–206. 20053163 10.2174/138620710790596736 PMC2908937

[25] Brennan FR, Morton LD, Spindeldreher S, Kiessling A, Allenspach R, Hey A, et al Safety and immunotoxicity assessment of immunomodulatory monoclonal antibodies. mAbs. 2010;2(3):233–55. 20421713 10.4161/mabs.2.3.11782 PMC2881251

[26] Oates J, Hassan NJ, Jakobsen BK. ImmTACs for targeted cancer therapy: Why, what, how, and which. Molecular immunology. 2015;67(2 Pt A):67–74. 25708206 10.1016/j.molimm.2015.01.024

[27] Linette GP, Stadtmauer EA, Maus MV, Rapoport AP, Levine BL, Emery L, et al Cardiovascular toxicity and titin cross-reactivity of affinity-enhanced T cells in myeloma and melanoma. Blood. 2013;122(6):863–71. 10.1182/blood-2013-03-490565 23770775 PMC3743463

[28] Cameron BJ, Gerry AB, Dukes J, Harper JV, Kannan V, Bianchi FC, et al Identification of a Titin-derived HLA-A1-presented peptide as a cross-reactive target for engineered MAGE A3-directed T cells. Science translational medicine. 2013;5(197):197ra03. 10.1126/scitranslmed.3006034 PMC6002776 23926201

[29] Purbhoo MA, Sutton DH, Brewer JE, Mullings RE, Hill ME, Mahon TM, et al Quantifying and imaging NY-ESO-1/LAGE-1-derived epitopes on tumor cells using high affinity T cell receptors. Journal of immunology. 2006;176(12):7308–16. 10.4049/jimmunol.176.12.7308 16751374

[30] McCormack E, Adams KJ, Hassan NJ, Kotian A, Lissin NM, Sami M, et al Bi-specific TCR-anti CD3 redirected T-cell targeting of NY-ESO-1- and LAGE-1-positive tumors. Cancer immunology, immunotherapy: CII. 2013;62(4):773–85. 10.1007/s00262-012-1384-4 23263452 PMC3624013

[31] Bossi G, Gerry AB, Paston SJ, Sutton DH, Hassan NJ, Jakobsen BK. Examining the presentation of tumor-associated antigens on peptide-pulsed T2 cells. Oncoimmunology. 2013;2(11):e26840 10.4161/onci.26840 24482751 PMC3894244

[32] Korzeniewski C, Callewaert DM. An enzyme-release assay for natural cytotoxicity. Journal of immunological methods. 1983;64(3):313–20. 6199426 10.1016/0022-1759(83)90438-6

[33] Raman MC, Rizkallah PJ, Simmons R, Donnellan Z, Dukes J, Bossi G, et al Direct molecular mimicry enables off-target cardiovascular toxicity by an enhanced affinity TCR designed for cancer immunotherapy. Scientific reports. 2016;6:18851 10.1038/srep18851 26758806 PMC4725365

[34] Bossi G, Buisson S, Oates J, Jakobsen BK, Hassan NJ. ImmTAC-redirected tumour cell killing induces and potentiates antigen cross-presentation by dendritic cells. Cancer immunology, immunotherapy: CII. 2014;63(5):437–48. 10.1007/s00262-014-1525-z 24531387 PMC11029007

[35] Thingnes J, Lavelle TJ, Hovig E, Omholt SW. Understanding the melanocyte distribution in human epidermis: an agent-based computational model approach. PloS one. 2012;7(7):e40377 10.1371/journal.pone.0040377 22792296 PMC3392240

[36] Neelapu SS, Tummala S, Kebriaei P, Wierda W, Gutierrez C, Locke FL, et al Chimeric antigen receptor T-cell therapy—assessment and management of toxicities. Nat Rev Clin Oncol. 2018;15(1):47–62. 10.1038/nrclinonc.2017.148 28925994 PMC6733403

[37] Topp MS, Gokbuget N, Stein AS, Zugmaier G, O'Brien S, Bargou RC, et al Safety and activity of blinatumomab for adult patients with relapsed or refractory B-precursor acute lymphoblastic leukaemia: a multicentre, single-arm, phase 2 study. Lancet Oncol. 2015;16(1):57–66. 10.1016/S1470-2045(14)71170-2 25524800

[38] Walker MR, Makropoulos DA, Achuthanandam R, Van Arsdell S, Bugelski PJ. Development of a human whole blood assay for prediction of cytokine release similar to anti-CD28 superagonists using multiplex cytokine and hierarchical cluster analysis. International immunopharmacology. 2011;11(11):1697–705. 10.1016/j.intimp.2011.06.001 21689786

[39] Stebbings R, Findlay L, Edwards C, Eastwood D, Bird C, North D, et al "Cytokine storm" in the phase I trial of monoclonal antibody TGN1412: better understanding the causes to improve preclinical testing of immunotherapeutics. Journal of immunology. 2007;179(5):3325–31. 10.4049/jimmunol.179.5.3325 17709549

[40] van den Berg CW, Okawa S, Chuva de Sousa Lopes SM, van Iperen L, Passier R, Braam SR, et al Transcriptome of human foetal heart compared with cardiomyocytes from pluripotent stem cells. Development. 2015;142(18):3231–8. 10.1242/dev.123810 26209647

[41] Kingsley E, Freeth J, Soden J. Specificity screening of antibodies and related molecules using human cell microarray technology. 13th Annual PEGS Summit; Boston. Boston2017.

