# Supplementary figures and images for: An approved in vitro approach to preclinical safety and efficacy evaluation of engineered T cell receptor anti-CD3 bispecific (ImmTAC) molecules
Source: PLoS One. 2018 Oct 15;13(10):e0205491. doi: 10.1371/journal.pone.0205491 (PMC6188753; doi:10.1371/journal.pone.0205491)

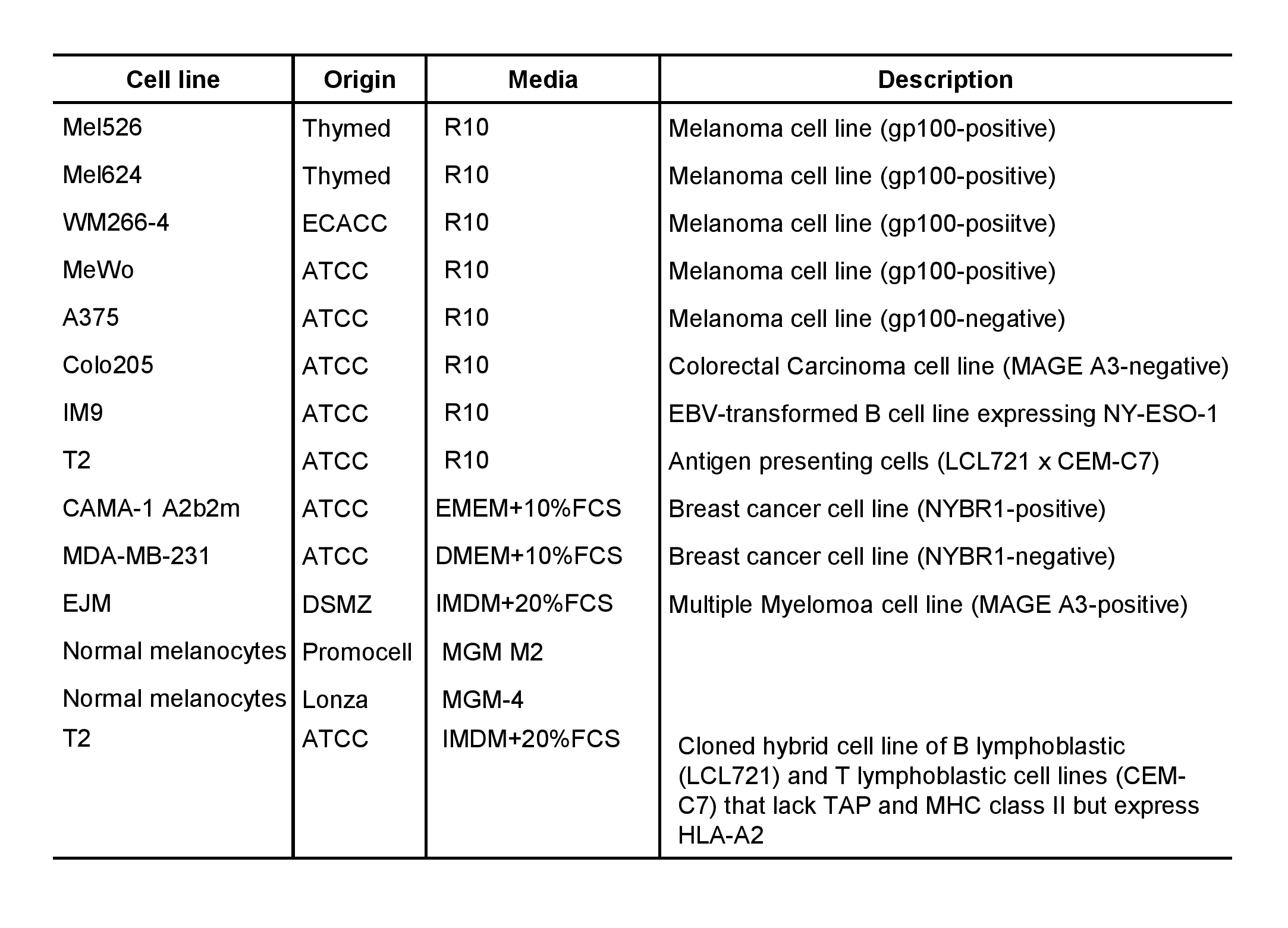

Supplement: S1 Table — R10; 10% RPMI, 1% FCS, 1% Penicillin/Streptomycin and 1% Glutamine. EMEM; Eagle’s Minimum Essential Medium. DMEM; Dulbecco’s Modified Eagles Medium. IMDM; Iscove’s Modified Dulbecco’s Media. MGM M2; serum-free, PMA-free optimal Melanocyte Growth Media. MGM-4; Melanocyte Growth Medium-4. (TIF) [file pone.0205491.s001.tif]

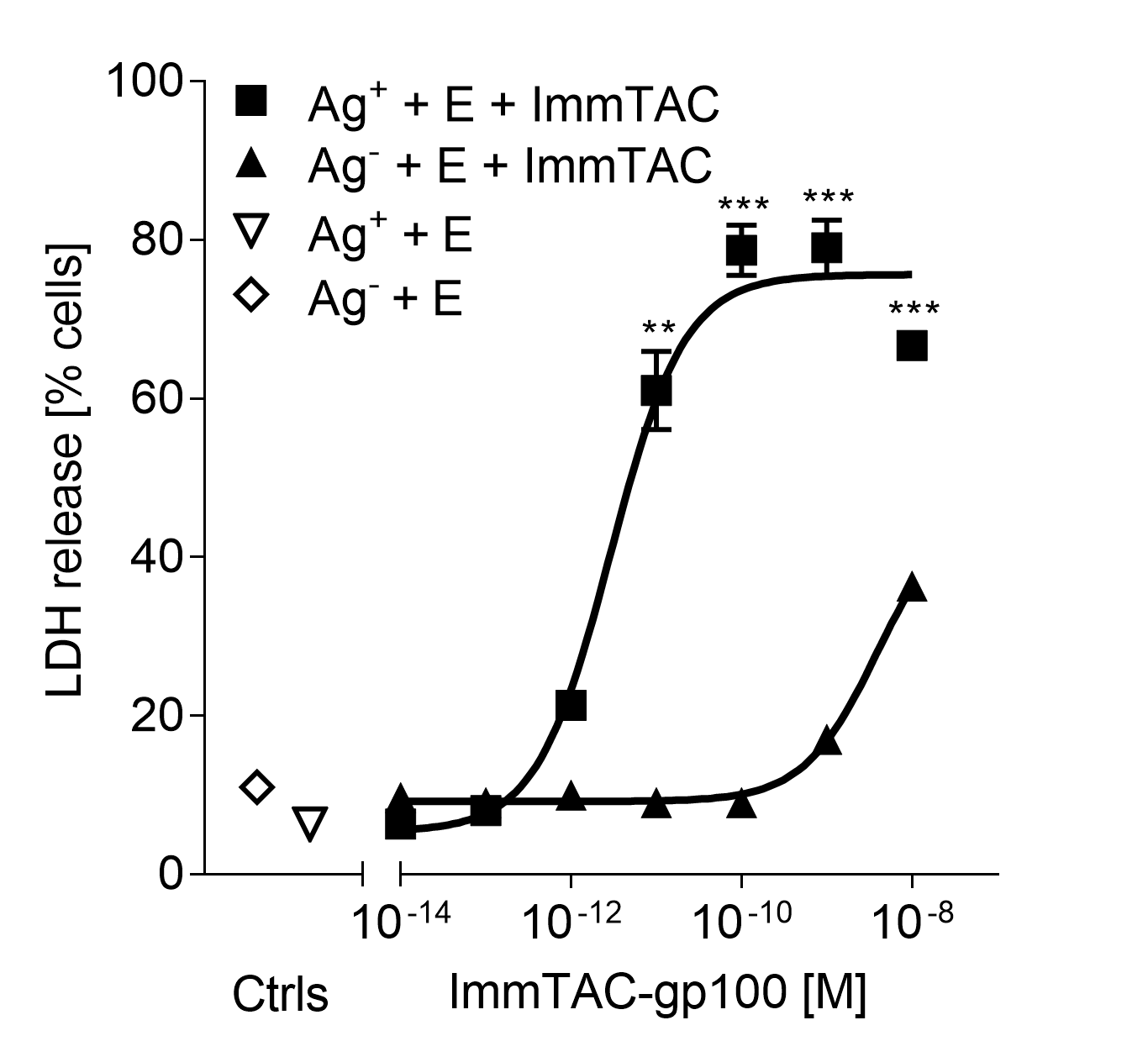

Supplement: S1 Fig — Ag+ cell lines (Mel526) that present target peptide-HLA and Ag- cell lines (A375) that do not present peptide-HLA were incubated with PBMC effector cells (E) from healthy donors in the presence or absence of ImmTAC-gp100 at increasing concentrations and LDH release was measured. Statistical difference between Ag+ and Ag- cells in the presence of effector cells (E) + ImmTAC was measured using a Two-way ANOVA with Sidak’s multiple comparison test where *** p<0.0001, **p<0.01. (TIF) [file pone.0205491.s002.tif]
